# EMT and Stemness in Tumor Dormancy and Outgrowth: Are They Intertwined Processes?

**DOI:** 10.3389/fonc.2018.00381

**Published:** 2018-09-12

**Authors:** Keren Weidenfeld, Dalit Barkan

**Affiliations:** Department of Human Biology and Medical Sciences, University of Haifa, Haifa, Israel

**Keywords:** tumor dormancy, epithelial mesenchymal transition, mesenchymal epithelial transition, cancer stem cells, disseminated tumor cells, metastasis, stemness, cancer recurrence

## Abstract

Metastases are the major cause of cancer patients' mortality and can occur years and even decades following apparently successful treatment of the primary tumor. Early dissemination of cancer cells, followed by a protracted period of dormancy at distant sites, has been recently recognized as the clinical explanation for this very-long latency. The mechanisms that govern tumor dormancy at distant sites and their reactivation to proliferating metastases are just beginning to be unraveled. Tumor cells, that survive the immune surveillance and hemodynamic forces along their journey in the circulation and successfully colonize and adopt to the new and “hostile” microenvironment and survive in a quiescent dormant state for years before emerging to proliferative state, must display high plasticity. Here we will discuss whether the plasticity of dormant tumor cells is required for their long-term survival and outgrowth. Specifically, we will focus on whether epithelial mesenchymal transition and acquisition of stem-like properties can dictate their quiescent and or their proliferative fate. Deeper understanding of these intertwining processes may facilitate in the future the development of novel therapies.

## Introduction

Metastasis is the spread of tumor cells from the primary site to distant organs and their subsequent growth, and is the major cause of cancer patient's mortality (1–5). Accumulating evidence in the literature suggest that metastasis can be an early event (6–11) and is not exclusive to late stage of tumor progression. Yet, it is well-recognized that metastasis is an inefficient process, given that only <0.02% of circulating tumor cells (CTCs) survive the immune surveillance and hemodynamic forces in the circulation (12). Surviving CTCs will colonize distant organs and become disseminated tumor cells (DTCs). Notably, the majority of DTCs do not survive the initial colonization, whereas those that survive may persist to reside in the secondary sites in a quiescent state (cellular dormancy) for many years (1, 13), or progressively grow to form metastases (2). This long-term survival and quiescence of the DTCs may account for the latent recurrence years and decades after primary tumor resection and adjuvant therapy (14). Hence, DTCs that will survive the initial steps of colonization at the distant organ must launch survival mechanisms allowing escape from apoptotic signals and long-term survival in their new and “hostile” microenvironment (non-permissive soil). Upon adequate signals arising in their microenvironment (permissive soil) these DTCs will switch from their quiescence state and launch cellular signals that will enable them to re-emerge to proliferative growth (1, 15). This is a testament of DTCs' high plasticity. In this review, we will discuss epithelial–mesenchymal plasticity of DTCs and their acquisition of stem cell-like properties as part of the mechanisms that will dictate whether they remain dormant or will re-emerge to metastatic outgrowth.

## Tumor dormancy and EMT plasticity

Epithelial to mesenchymal transition (EMT) occurs during gastrulation and neural crest formation of the developing embryo (16, 17) and in pathological conditions such as wound healing and metastasis (18, 19). Loss of cell polarity and epithelial markers such as the epithelial adhesion protein E-cadherin and cytokeratin 18, and gain of mesenchymal markers such as vimentin and fibronectin are the hallmarks of EMT. EMT was shown to facilitate invasive and high motility characteristic of tumor cells, thus enabling their dissemination from the primary site (19, 20). Notably, primary tumor heterogeneous nature (21) is also demonstrated by EMT occurrence only within a subpopulation of cells within the primary tumor, usually at the leading edge of the tumor (19, 22, 23). Those tumor cells at the leading edge undergoing EMT will initiate their journey in the circulation by successfully invading through the basement membrane. This can be facilitated by their reduced apical-basal polarity and epithelial adhesion proteins.

Snail, Slug, Zeb1 and Twist1 are some of the transcription factors that repress epithelial adhesion protein such as E-cadherin (EMT-TF) and were shown to orchestrate EMT programming (24, 25). However, several reports demonstrate that while EMT-TF expression is required for dissemination, their repression is required to promote metastatic growth *in vivo*. Tsai and colleagues (26) demonstrated that while expression of Twist1 is required for EMT and tumor dissemination at distant sites, Twist1 repression was indispensable for DTCs outgrowth (26). Similarly, repression of homeobox factor Prrx1 (inducer of EMT) was central for the development of metastasis *in vivo* (27). Snail expression in breast cancer metastasis was shown to be transient, whereas forced and prolonged expression of Snai1 decreased lung metastasis (28). Hence, mesenchymal-like DTCs may remain in a dormant state after colonizing the distant organ (27, 29–32), whereas metastatic growth may be dependent on DTCs ability to regain back their epithelial phenotype by mesenchymal epithelial transition (MET) (19, 26, 29, 31, 33). Importantly, these findings are consistent with clinical observations demonstrating epithelial phenotype of human metastases resembling the primary tumor (34). Hence, high plasticity of DTCs is required for their ability to transition between epithelial to mesenchymal and back to their epithelial state during the different steps in the metastatic process.

## EMT and acquisition of stem-like properties

Activation of the EMT program, eliciting dissemination of cancer cells to distant organs, can also bestow these cells with high plasticity via acquiring stem-like traits. According to the model of cancer stem cells (CSC), a small subpopulation of cancer cells is endowed with stem like-traits with the potential to promote cancer progression. These CSC attain tumor-initiating and metastatic potential, while the non-CSCs lack these traits (35).

Several studies have demonstrated this link between EMT, stemness, and the metastatic initiating potential of DTCs. Induction of EMT in transformed epithelial cells was shown previously to culminate in endowing cells with stem-like traits (36, 37). These stem-like traits in transformed epithelial cells promoted the initiation of primary tumors and accelerated metastasis (19, 36, 38, 39).

The link between undifferentiated status, stemness, and dissemination of tumor cells from the primary site was also demonstrated. Several studies report how EMT-TF Zeb1 can promote stemness and inhibit epithelial differentiation by repressing miR-200 family members (33, 40, 41). In addition, GATA3, a transcription factor known to determine cell fate of luminal epithelial cells, was shown to be lost during early stages of malignant progression in the MMTV-PyMT mouse model. This loss was followed by cell dissemination of CSC-like cells (42, 43). Metastatic progression of lung adenocarcinoma in mouse models was shown to be associated with a stemness program, mediated by loss of Nkx2-1 expression (42, 44, 45). Hence, these observations suggest that CTC arriving to distant organs may already be endowed with CSC-like traits. Notably, Malanchi and colleagues previously demonstrated that only the CSC population of DTCs was capable to initiate metastatic nodules at secondary site (46).

Overall, these studies suggest that EMT, along with the resulting acquisition of stem cell-like properties, facilitate dissemination and consequently the outgrowth of DTCs at distant organs (47).

Intriguingly, several studies in breast cancer cells have identified a sub-population of non-CSCs that are highly plastic and can switch to CSC state (48–50). This transition can be attributed to a stochastics event (50) or can be driven by the metastatic niche (51).

Microenvironmental cues which are part of the metastatic milieu, such as TGF-β, was shown to induce plastic basal-like CD44^low^ breast cancer cells to acquire a CSC-like phenotype via chromatin remodeling at the ZEB1 promoter (49). In line of these previous findings, Weidenfeld K et al. recently demonstrated that expression of LOXL2 endowed dormant DTCs with CSC-like traits eliciting their transition to metastatic outgrowth. These stem-like traits were dependent on EMT and were driven by hypoxia (52). These findings are in line with a previous report demonstrating the role of EMT in the switch from tumor dormancy to proliferative growth (53). Hence, non-CSC residing at distant organs can remain dormant until appropriate signals will endow them with stem-like properties and reactivation. Indeed, breast cancer DTCs were shown to activate the stromal cells in their vicinity to release niche extracellular matrix (ECM) components such as Periostin and Tenacin C. These ECM components in turn activated tumorigenic stem cell signaling pathways such as Wnt, Nanog, and Oct4 in the residing DTCs, leading to their metastatic outgrowth (46, 47). Microenvironmental cues associated with inflammation were recently shown to also promote dormant DTCs outgrowth. Inflammation of the lung induced EMT of DTCs via the expression of Zeb1, resulting in the reactivation of the quiescent DTCs (54). Moreover, the formation of a fibrotic-like milieu enriched with type I collagen and its cross linking by LOX was previously shown to promote the transition of dormant DTCs to metastatic outgrowth (55, 56). Of note, matrix stiffening is induced by increased Col-I deposition and its cross-linking. Mechanical stiffness of the matrix was shown to regulate EMT via Twist 1 (57) and promote CSC-like traits of cancer cells (58).

Hence, changes in the ECM components and mechanical compliance may provide a “fertile soil” to promote dormant DTCs plasticity and outgrowth (15).

## Tumor dormancy and stemness

Adult stem cells are undifferentiated cells, residing in tissue in a quiescent state until signals arising in their surrounding niche will direct them to self-renew and differentiate to yield some or all of the major specialized cell types of the tissue. The link between dormant DTCs and their acquisition of stem-like traits has been proposed. DTCs residing at distant organ are exposed to un-permissive “soil.” In order to survive and grow, these cells will launch some intrinsic dormant programs inhibiting self-renewal and inducing cell cycle arrest and survival pathways [Quiescent stemness; (51, 59)]. Indeed, DTCs in bone marrow detected in early stage of breast cancer patients were shown to display a putative stem-like phenotype (60). In addition, DTCs of prostate cancer cells, recovered from bone marrow, were significantly enriched for a CSC phenotype (61). Importantly, the transition of these DTCs to CSCs was regulated by niche-derived Growth Arrest Specific 6 (GAS6), previously shown to regulate dormancy (62). GAS6 activated mTOR signaling in the prostate cancer DTCs through the Mer receptor, endowing them with cancer stem-like traits (61). Similarly, an orphan nuclear receptor NR2F1 expression was shown recently to be upregulated in DTCs from prostate cancer patients carrying dormant disease (63). NR2F1 induced quiescence was dependent on Retinoic Acid Receptor Beta (RARβ) and cyclin dependent kinase (CDK) inhibitors and the stem cell factor SOX9 (63). Additionally, Bone Morphogenetic Protein-7 (BMP- 7) secreted from bone stromal cells induces senescence in prostate CSC by activating Bone Morphogenetic Protein Receptor Type II (BMPR2)-p38-NDRG1 axis. Notably, this BMP-7-induced senescence in CSCs was reversible upon withdrawal of BMP-7 (64). Another regulator of stem cell activity, leukemia inhibitory factor (LIF), was shown to promote tumor dormancy of breast cancer cells in the bone. Loss of LIF receptor (LIFR) of breast cancer DTCs in turn promoted their outgrowth from quiescence and down-regulated CSC associated genes (65).

These findings suggest that dormant DTCs might retain stem-like properties such as quiescence, yet will shift to self-renewal program upon cues from their niche, leading to their reactivation.

Overall, several studies support the role of EMT and CSC-like traits in promoting tumor dormancy and MET during metastatic outgrowth. However, EMT and CSC-like traits have been shown, in other studies, to inhibit tumor dormancy. These conflicting findings as reviewed above suggest high plasticity in the EMT and acquisition of CSC-like states during the transition from tumor dormancy to metastatic outgrowth.

## DTCs fluctuation between EMT–met states and CSC-like traits

**Scheme 1:**
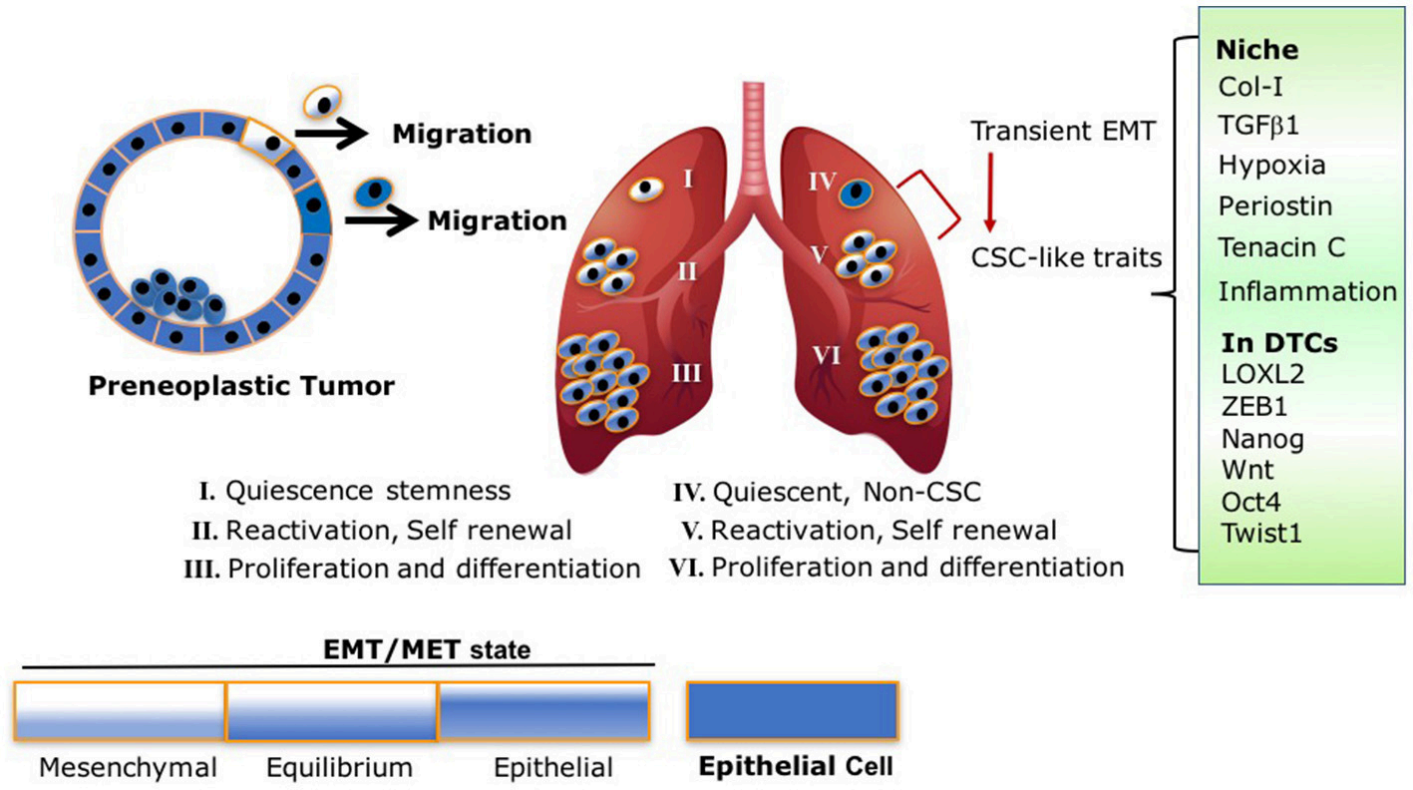
EMT/MET transient state and CSC traits are intertwined processes: following model illustrates the different fluctuations between the EMT/MET states of early disseminated DTCs and their link to stemness and metastatic outgrowth. **(I)** Dormant DTCs with EMT/MET state lining toward more mesenchymal phenotype activate quiescence stemness. **(II)** DTCs with EMT/MET at “equilibrium” are highly plastic with self-renewal capacity resulting in the establishment of micrometastases. **(III,VI)** Macrometastases with EMT/MET state lining to epithelial phenotype proliferate and differentiate. **(IV,V)** Non-CSC **(IV)** undergo transient EMT endowing the cells with CSC-like traits and self-renewal capacity resulting in the establishment of micrometastases **(V)**. This transition is mediated by signals arising at their niche (Col-I enriched fibrotic milieu, TGFβ1, Periostin, Tenancin C, inflammation, hypoxia) which in turn can activate accordingly EMT programs via expression of EMT-TF and/or LOXL2 resulting in acquisition of CSC-like traits.

Intriguingly, a recent study by Harper et al. demonstrated EMT plasticity within Her2+ DTCs. Early disseminated Her2+ DTCs underwent EMT, expressed the epithelial marker CK8/18+, and retained prolonged dormancy in the bone marrow and lungs. These dormant DTCs eventually developed metastases (7). These findings suggest that a partial EMT is ample for early dissemination, dormancy and outgrowth (7). Indeed, several clinical reports demonstrate the presence of partial EMT or a hybrid EMT/MET CTC (66–68). Furthermore, many cancer cells may metastasize without completely losing their epithelial phenotype and or completely gaining mesenchymal traits (69, 70). Hence, EMT is not an “all-or-none” response, rather it involves transitional states (71, 72), which can reconcile the accumulating experimental and clinical evidence demonstrating EMT of DTCs and MET in proliferating metastases (19, 26, 29, 31, 33) Scheme 1.

Therefore, based on these previous findings and recent reports, we propose the following model Scheme 1 that may reconcile the overall findings. As illustrated in Scheme 1, early DTCs, which account for the source of recurring cancers, may exist in a transient EMT/MET state as was previously proposed (72). Dormancy of the early DTCs may display an EMT/MET transient state leaning to a more mesenchymal phenotype, resulting in CSC-like traits responsible for their quiescence. Initial induction of DTC outgrowth by ECM remodeling and other signals arising at the metastatic site may tilt the EMT/MET toward an “equilibrium” state, which may endow the cells with the highest plasticity to initiate self-renewal of the cells. As the EMT/MET state progressively leans to a more epithelial phenotype, this in turn will increase cell proliferation and differentiation of the growing macrometastases. Importantly, key elements in the proposed model have been supported by a recent analysis of CSC markers in human metastatic breast cancer cells (31). Furthermore, breast cancer stem cells were reported to exist in distinct EMT and MET states characterized by expression of distinct CSC markers. Notably, breast cancer cells with dual expression of both sets of markers were demonstrated to have the highest degrees of plasticity (32). Hence, the transient EMT/MET gradient state linked to CSC-like traits may dictate whether DTCs will remain dormant or emerge to metastatic outgrowth. Importantly, we should also consider the other scenario where DTC can promote dormancy programs at distant sites independent from the acquisition of stem-like properties. These DTCs however, will acquire stem-like properties mediated by a transient EMT leading to their reactivation. This transition may be driven by signals in their metastatic microenvironment Scheme 1.

The ability of DTCs to fluctuate between EMT–MET states and acquire different CSC-like traits can also facilitate their immune evasion during metastasis (73). Several EMT-TF such as Twist1 and Zeb1 were shown to have immunosuppressive functions. Zeb1, by downregulating miR-200s, promoted PD- L1 upregulation. PD- L1 is an immune checkpoint regulator of CD8^+^ T cells (74). Melanoma cells expressing another EMT-TF known as Snail, induced immune suppression via activation of regulatory T cells and impaired dendritic cell activity (75). In addition, Mesenchymal-like DTCs, which often have elevated expression of TGF-β, may escape attack by cytotoxic CD8+ T cells (76). Similarly, activation of CSC-like traits in DTCs such as expressing Sox-dependent stem-like state, followed by actively silencing WNT signaling, can promote quiescence of DTCs and their immune evasion (77).

Overall, the fluctuation of DTCs between EMT-MET states and their acquisition of different CSC-like traits will enable their adaption to the distant site and their evasion of the immune system.

## Concluding remarks

Dormant DTCs residing at distant sites must display high plasticity to successfully overcome the un-permissive “soil” and emerge to metastatic growth. The plasticity of residing DTCs due to acquiring a partial EMT phenotype is an emerging concept that is supported by recent clinical data. This in turn can promote different CSC-like traits during the different steps in metastatic progression. The scope of this review was to reveal the potential link between EMT/MET and CSC-like traits in the transition of dormant DTCs to metastatic outgrowth. Several lines of evidence presented in this review suggest that EMT and stem cell-like traits are intertwined processes dictating DTCs fate. These intertwined processes are highly complex and warrant additional research in order to utilize these emerging concepts in our battle against cancer recurrence.

## Authors contributions

KW: research and writing, DB: conception, research, writing, and editing.

### Conflict of interest statement

The authors declare that the research was conducted in the absence of any commercial or financial relationships that could be construed as a potential conflict of interest.
